# Systematic review of the effects of the intestinal microbiota on selected nutrients and non-nutrients

**DOI:** 10.1007/s00394-017-1546-4

**Published:** 2017-10-30

**Authors:** Colette Shortt, Oliver Hasselwander, Alexandra Meynier, Arjen Nauta, Estefanía Noriega Fernández, Peter Putz, Ian Rowland, Jonathan Swann, Jessica Türk, Joan Vermeiren, Jean-Michel Antoine

**Affiliations:** 1 Johnson & Johnson EAME, Foundation Park, Maidenhead, SL6 3UG UK; 2 DuPont Nutrition and Health, c/o Danisco (UK) Ltd., 43 London Road, Reigate, Surrey, RH2 9PW UK; 3 Mondelēz France R&D, 6 Rue René Razel, 91400 Saclay, France; 4 FrieslandCampina, Stationsplein 4, 3818 LE Amersfoort, The Netherlands; 5 Nofima Hovedkontor, Muninbakken 9-13, Breivika Postboks 6122, Langnes, 9291 Tromsø, Norway; 6 University of Applied Sciences, FH Campus Wien, 1100 Vienna, Austria; 7 Department of Food and Nutritional Sciences, University of Reading, Whiteknights, Reading, RG6 6AP UK; 8 Imperial College London, South Kensington Campus, London, SW7 2AZ UK; 9 Yakult Germany, Forumstraße 2, 41468 Neuss, Germany; 10 Cargill R&D Centre Europe, Havenstraat 84, 1800 Vilvoorde, Belgium; 11 Danone Research, Route Départementale 128, 91767 Palaiseau Cedex, France

**Keywords:** Intestinal microbiota, Metabolism, Metabolites, Dietary substrates

## Abstract

**Purpose:**

There is considerable interest in the effects of the intestinal microbiota (IM) composition, its activities in relation with the metabolism of dietary substrates and the impact these effects may have in the development and prevention of certain non-communicable diseases. It is acknowledged that a complex interdependence exists between the IM and the mammalian host and that the IM possesses a far greater diversity of genes and repertoire of metabolic and enzymatic capabilities than their hosts. However, full knowledge of the metabolic activities and interactions of the IM and the functional redundancy that may exist are lacking. Thus, the current review aims to assess recent literature relating to the role played by the IM in the absorption and metabolism of key nutrients and non-nutrients.

**Methods:**

A systematic review (PROSPERO registration: CRD42015019087) was carried out focussing on energy and the following candidate dietary substrates: protein, carbohydrate, fat, fibre, resistant starch (RS), and polyphenols to further understand the effect of the IM on the dietary substrates and the resulting by-products and host impacts. Particular attention was paid to the characterisation of the IM which are predominantly implicated in each case, changes in metabolites, and indirect markers and any potential impacts on the host.

**Results:**

Studies show that the IM plays a key role in the metabolism of the substrates studied. However, with the exception of studies focusing on fibre and polyphenols, there have been relatively few recent human studies specifically evaluating microbial metabolism. In addition, comparison of the effects of the IM across studies was difficult due to lack of specific analysis/description of the bacteria involved. Considerable animal-derived data exist, but experience suggests that care must be taken when extrapolating these results to humans. Nevertheless, it appears that the IM plays a role in energy homeostasis and that protein microbial breakdown and fermentation produced ammonia, amines, phenols and branch chain fatty acids, and a greater diversity in the microbes present. Few recent studies appear to have evaluated the effect of the IM composition and metabolism per se in relation with digestible dietary carbohydrate or fat in humans. Intakes of RS and prebiotics altered levels of specific taxa that selectively metabolised specific prebiotic/carbohydrate-type substances and levels of bifidobacteria and lactobacilli were observed to increase. In controlled human studies, consistent data exist that show a correlation between the intake of fibre and an increase in bifidobacteria and short-chain fatty acids, in particular butyrate, which leads to lower intestinal pH. Dietary polyphenols rely on modification either by host digestive enzymes or those derived from the IM for absorption to occur. In the polyphenol-related studies, a large amount of inter-individual variation was observed in the microbial metabolism and absorption of certain polyphenols.

**Conclusions:**

The systematic review demonstrates that the IM plays a major role in the breakdown and transformation of the dietary substrates examined. However, recent human data are limited with the exception of data from studies examining fibres and polyphenols. Results observed in relation with dietary substrates were not always consistent or coherent across studies and methodological limitations and differences in IM analyses made comparisons difficult. Moreover, non-digestible components likely to reach the colon are often not well defined or characterised in studies making comparisons between studies difficult if not impossible. Going forward, further rigorously controlled randomised human trials with well-defined dietary substrates and utilizing omic-based technologies to characterise and measure the IM and their functional activities will advance the field. Current evidence suggests that more detailed knowledge of the metabolic activities and interactions of the IM hold considerable promise in relation with host health.

## Introduction

**Fig. 1:**
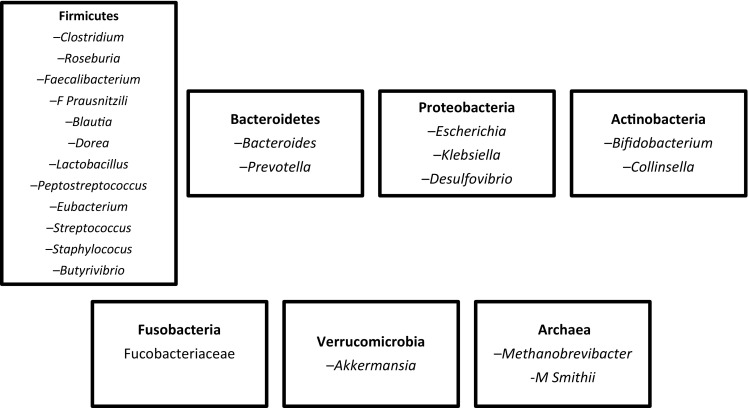
Main phyla and genera with selected examples of human gut microbiota

It is well-recognised that humans are colonised by trillions of microbes, with an estimated microbial metagenome of at least 100-fold that of human cells and that these microbes possess a far greater repertoire of metabolic and enzymatic capabilities than their human hosts [1–3]. The IM contribute significantly to human health and may play a role in the development and/or prevention of certain non-communicable diseases. Thus, in relation with nutrition research, there is considerable interest in the functional interactions between the IM, ingested dietary substrates, and host metabolism [4–7]. While there is considerable IM diversity, a few phyla predominate: Bacteroidetes, Firmicutes, Actinobacteria, Proteobacteria, Verrucomicrobia, Fusobacteria, and a limited number of Archaea, mainly methanogens (Fig. 1). In addition, despite the consistency of these major phyla, their relative proportions and the species present can vary dramatically between individuals.

These commensal microbes play a major role in the breakdown, absorption, and metabolism of key dietary constituents, contributing, for example, to the breakdown of indigestible dietary substrates, vitamin biosynthesis, transformation of compounds to bioactives, and the production of various gases as by-products of fermentation. Core functions of the IM include pathways associated with carbohydrate and amino acid fermentation. However, not all pathways are represented in the core and variable functions are sometimes restricted to specific species or strains, including, for example, vitamin and drug synthesis, metabolism, and nutrient transport. A key challenge is to understand which members of the microbial community have similar functionalities and can substitute for one another. The IM metabolism extends the host’s metabolic capacity through, for example, substrate hydrolysis, reduction, and methylation. A more thorough understanding of the role the IM plays in the metabolism of dietary substrates and of the pertinent and dominant members of the IM and community profiles hold considerable promise to facilitate the development of novel dietary strategies. This systematic review considers the complex interplay and metabolic effects of the IM on energy homeostasis and a selection of candidate dietary substrates: protein, digestible carbohydrates, fat, fibres, RS, and polyphenols. During the review, particular attention was paid to the resulting metabolites and the characterisation of the IM which was predominantly implicated in each case.

## Methods

**Fig. 2:**
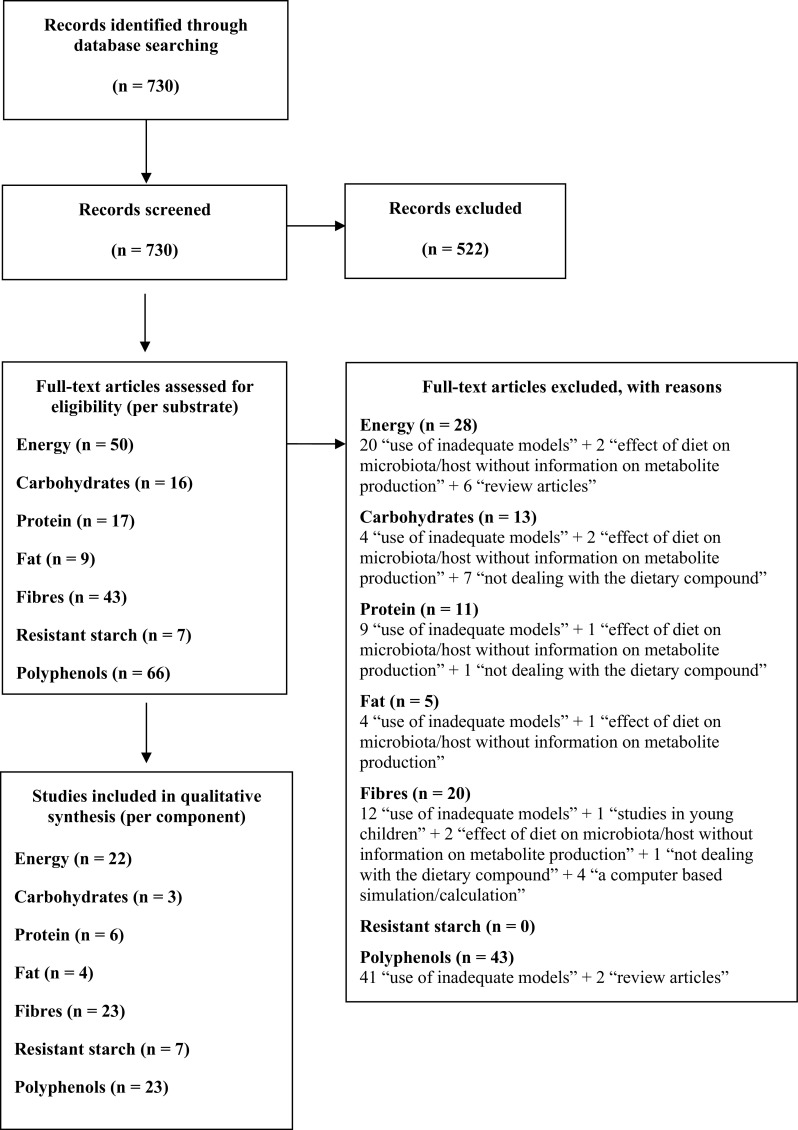
Search phases of the systematic review

A systematic search of the literature in PubMed was undertaken to identify relevant human and animal studies. Publications in English, between March 2005 and 2015, were retrieved using a combination of MESH terms and keywords (metabolism or breakdown, degradation, fermentation, digestion, transformation, bioavailability, absorption: microbiota or bacteria, flora, and microbiome) to identify original research examining the effect of the IM on the metabolism of energy, protein, fat, digestible carbohydrates, fibres, RS, and polyphenols (isoflavones, phenolic acids, and flavonoids). Details of the search can be accessed via http://www.crd.york.ac.uk/PROSPEROFILES/19087_STRATEGY_20161106.pdf. No exclusion criteria were applied in relation with health status of the study populations. However, general reviews, studies involving children less than 15 years, studies using certain models (ruminants, birds, and ileostomised subjects), and in vitro data were excluded from the assessment. Studies dealing with the impact of the dietary substrates on the IM composition alone or effects on the host as well as studies not including an analysis of the IM were also excluded in the course of the screening of the abstracts and papers unless metabolites were measured. The process applied for literature selection is summarised in Fig. 2. Each abstract and subsequently each selected paper was screened independently for eligibility by two individuals. In the case of disagreement, consensus was reached by discussion. The review followed the approach of the Preferred Reporting for Systematic Reviews and Meta-Analysis Guidelines [8]. Details of the systematic review were registered in the PROSPERO International Prospective Register of Systematic Reviews (registration number CRD42015019087) (http://www.crd.york.ac.uk/PROSPERO) and selected details of the studies included in the review are provided. During the systematic evaluation, study data were reviewed, where possible, in relation to substrate disappearance, appearance of metabolites, changes in the IM composition, and any reported metabolic consequences in human and animal studies, respectively.

## Results

### Impact of the intestinal microbiota on energy homeostasis

The IM has coevolved with the human host to perform a number of functions that would otherwise not be accomplished without the IM. Data from studies involving humans and germ-free (GF), lean, and obese animals suggest that the IM plays an important role in the assimilation, storage, and expenditure of energy obtained from dietary substrates. For example, while interacting symbiotically with each other and with the intestinal environment, members of the IM break down non-digestible polysaccharides, producing monosaccharides and short-chain fatty acids (SCFA), and allow the host to salvage energy from otherwise indigestible dietary substrates. The IM also affects energy balance through metabolites it produces by regulating gene expression. Overall, 22 publications were identified in relation with the IM and energy homeostasis, and of these, nine human [9–17] and 13 animal [18–30] studies were reviewed. In the human studies, the number of subjects varied from 12 to 80 and the study duration varied from 1 day to 6 months. Of the human studies, five involved normal weight subjects, three involved obese subjects, and one compared lean and obese subjects.

### Data from human studies relating to energy homeostasis

Studies addressing the role of the IM in the host energy homeostasis have assessed energy either as the amount of energy in faeces measured by bomb calorimetry (energy that has not been used either by the host or by the microbes), a change in body mass index (BMI, kg/m^2^), and/or changes in weight. Change in the amount of SCFA in faeces was also recorded, though it should be noted that the role of faecal SCFA in energy homeostasis has yet to be fully clarified. The effect on the IM in healthy humans on a regular diet was compared with the same diet either devoid or supplemented with a formula with 14 g/l of pea fibre and fructo-oligosaccharide (FOS) [10]. The fibre-free diet was associated with a decline in all microbial species and total SCFA. Butyrate was the only SCFA reduced during the fibre-supplemented diet and this reduction was correlated with a proportional decrease of *Faecalibacterium prausnitzii* [10]. This was the first study to show a strong positive correlation between the proportion of *F. prausnitzii* and that of butyrate in individuals on a normal diet and that the reduction in *F. prausnitzii* on switching to a fibre-free or fibre-supplemented diet correlated with the reduction in faecal butyrate. It has also been shown that a change in composition of the IM was associated with a change in energy lost in faeces in lean individuals; a 20% increase in *Firmicutes* and a corresponding decrease in *Bacteroidetes* increased energy harvest by 150 kcal [12]. IM metabolism was also explored following sleeve gastrectomy and weight loss. Butyrate production was reduced and correlated with a decrease in butyrate-producing microbes *Coprococcus* spp., *Eubacterium rectale*, *Ruminococcus obeum,* and *Lachnospiraceae*. In addition, excretion of non-esterified fatty and bile acids was increased and correlated with an increase in the ratio of Bacteroidetes/Firmicutes; there was also an increase in *B. vulgatus,* and *Bacteroides* spp., *F. prausnitzii*, and a decrease of Firmicutes: *Clostridium, Eubacterium, Dorea,* and *Coprococcus* spp. The authors highlighted that the energy-reabsorbing potential of the IM decreased following gastrectomy as indicated by the Bacteroidetes/Firmicutes ratio [13]. Others have reported a decreased concentration of *Clostridium histolyticum, C. lituseburense, E. rectale/C. coccoides*, and increased concentration of *Bacteroides/Prevotella* was associated with a significant increase in weight loss [14]. Rahat-Rozenbloom reported an increased production of colonic SCFA correlated with a higher concentration of Firmicutes, as well as a higher ratio of *Firmicutes/Bacteroides* in obese compared to lean individuals and suggested that the effect of diet and colonic flora on faecal SCFA in lean and obese warrants further attention [15]. Ross and colleagues compared urinary and faecal metabolites after either a refined or a whole grain (WG) diet [16]. The WG diet induced an increase in faecal acetate and butyrate, and a decrease in isovalerate and succinate which correlated with a decrease in *C. perfringens, C. difficile* and an increase in *C. leptum*. Others have explored the relationship between the IM and BMI in a cohort of monozygotic twins [17]. The IM of the cohort of monozygotic twins divided into a ‘low BMI’ and a ‘high BMI’ sibling group showed that at the phylum level, *Bacteroides/Firmicutes* ratio was similar, and at the group level, *Clostridium* cluster IV diversity decreased with increasing BMI. At the genus level, *E. ventrosium, R. intestinalis,* and *E. rectale* were more abundant in the higher BMI group, while more plant degrading microbes, *O. guilliermondi*, *C. cellulosi, R. bromii,* and *S. termitidis* were more abundant in the ‘low BMI’ group which also had lower faecal SCFA concentrations. The authors suggested that the differences in IM and fermentation patterns could have affected energy homeostasis.

### Data from animal studies relating to energy homeostasis

When mice with different IM (a conventional control, a simplified human model with and without *C. ramosum* and *C. ramosum* alone) were used in a comparison test [19], mice harboring *C. ramosum*, a member of the Erysipelotrichi class within the Firmicutes phylum, alone or with seven other species, gained more weight and body fat and displayed a higher feed efficiency than the group without *C. ramosum*. These effects were associated with an increased gene expression of a glucose transporter and an epithelial fatty acid translocase, leading to enhanced bioavailability. Romo-Vaquero and colleagues [23] modulated the IM of lean and obese Zucker rats with a Rosemary extract and reported a higher SCFA content in faeces from obese compared to lean rats. In lean rats, a specific increase in the level of *Bifidobacterium* genus and reduction of *C. leptum* group correlated with a significant reduction of SCFA. However, the increase in *B. coccoides, Bacteroides/Prevotella*, and decrease in *Lactobacillus/Leuconostoc/Pediococcus,* did not change the percentage of proteins, fat, fibre, or carbohydrates excreted in faeces. A correlation between a fibre/RS induced IM change, an increase of bifidobacteria, a decrease in *Bacteroides/Prevotella, Porphyromonas*, and *Enterococcus*, and an increase in colonic SCFA and branch chain fatty acids (BCFA), was also reported in rats fed diets containing red meat [24]. Others have used dietary RS to increase the caecal concentration of SCFA producing microbes, *Bacteroides* spp., *Bifidobacterium* spp., *Lactobacillus* spp., and *Clostridium* clusters IV and XIV [32] and reported an increase in SCFA concentration. Based on tests in an n-3 polyunsaturated fatty acids depleted mouse model [25], an increase in body weight was reported after a FOS induced increase of B*ifidobacterium* and *Bacteroides* without an increase in food intake. At the same time, they reported an increase in confounding mechanisms, such as an increase in glucagon-like peptide (GLP-1). This increase in body weight following FOS intake is in direct contrast to many studies showing a reduction in weight following prebiotic intake. The authors suggested that FOS promoted body weight gain in the n-3 depleted mice by increasing energy efficiency through increasing lactobacilli and counteracting the catabolic status induced by n-3 polyunsaturated fatty acid depletion. Others have demonstrated [22] that colonizing GF mice with *B. thetaiotaomicron* and *M. smithii* nearly doubles their daily weight gain, and increases fat pad weight by 50%. Firmicutes were shown to increase bioavailability of fatty acids by increasing the number of lipid droplets in the intestinal epithelium in a Zebra fish model and the authors concluded that different members of the IM may affect fatty acid absorption via distinct mechanisms [31].

Until now, there have been relatively few human studies of long duration that explored the role of the IM in relation with energy homeostasis and results in animals need to be interpreted with caution. The complexity of bacterial classification and the varied analytical methods used also add challenges in interpreting results in relation with energy homeostasis: for example, within an order like Clostridiales, different species may have completely different effects; therefore, specificity is required to fully interpret effects. Many studies also report various effects of dietary intakes without fully defining composition or chemical structure of specific substrates that impact on SCFA production, such as undigested carbohydrates or fibre. This makes comparing relative SCFA production very difficult when we cannot control precisely for quantities of substrate present. In addition, it is also difficult to relate faecal SCFA, with either SCFA production or energy availability to the host, from IM activity without concomitant or dynamic measures of plasma SCFA. Unfortunately, such dynamic blood SCFA flux experiments following food ingestion are few though warranted to increase understanding particularly in relation with energy homeostasis.

### Impact of the intestinal microbiota on the metabolism of macro-nutrients

A significant proportion of dietary carbohydrates, proteins, and all fats are absorbed before entering the large intestine. However, if it were not for the IM, the indigestible carbohydrates and proteins reaching the colon would be eliminated in the stool without further absorption. Few recent human studies have investigated the impact of the IM on the bioavailability and/or the bacterial metabolism of macro-nutrients (digestible carbohydrates, proteins, and fat) as they are largely absorbed in the early part of the gut and modifications observed seem to reflect the balance between these three digestible macro-nutrients. For digestible carbohydrates, no study in humans was identified that met our inclusion criteria, therefore, only data on animal models are presented [33–35]. For proteins, we identified six publications [34–39]. Of these, one was conducted in humans involving 11 subjects [38] and five involved animal models [34–37, 39]. For fats, one study conducted in humans with 15 subjects [40] and three in animals were identified [21, 41, 42].

### Data from animal studies on digestible carbohydrates

In a study on Beagle dogs, metabolites produced following 3 weeks of a control diet (providing crude proteins and RS) or a high-starch diet or a high-protein diet were compared [34]. Higher total SCFA was observed with the high-starch and control diets compared to the high-protein diet. The high-starch diet led to lower concentrations of faecal valerate, BCFA, and ammonia concentrations compared to the high-protein diet. Another study investigated the effect of four different diets in pigs with a T-cannula at the distal ileum: in particular, a control diet based on corn and soybean meal or three diets containing 75% of the control diet and 25% being replaced by ligno-cellulose, apple pectin, or corn starch [35]. The starch diet induced a two-fold higher total SCFA change compared to cellulose and apple pectin-based diet in the ileum and two-fold less total SCFA change compared to the apple pectin diet only in the faeces. There was a lower ileal pH following the apple pectin diet, whereas the cellulose-containing diet increased faecal pH. No significant difference was observed in the ileal and faecal SCFA concentrations between the starch and control diets. The differences observed between the starch diet and both the cellulose and apple pectin diets can be explained by the fact that starch is considered an easily fermentable source of carbohydrates in the small intestine which results in higher ileal concentrations of SCFA compared to pectin and cellulose and that this effect disappears in the distal parts of the intestine due to the higher digestibility of starch resulting in it either not reaching the colon or doing so only in small amounts. Ileal and faecal phosphorus recovery was also reduced after the cellulose- and starch-based diets. The starch diet increased the net phosphorus absorption from the large intestine compared to the cellulose and pectin-based diets. On the other hand, cellulose and pectin-based diets tended to cause a net phosphorus secretion into the large intestine. These differences in phosphorus absorption may reflect different bacterial requirements for the fermentation of the different carbohydrates tested. Indeed, it is known that degradation of cellulose and hemicellulose requires more phosphorus than the degradation of starch [43]. The effect of diets with differing ratios of digestible and RS was investigated in rats [33]. They were provided with diets containing 25% of casein and 0, 10, 20, 30, or 40% of high-amylose maize starch (HAMS, which is high in RS in place of corn starch) for 4 weeks [33]. The mucus layer thickness increased with increasing amount of HAMS in the diet and caecal total SCFA, acetate and propionate were significantly increased when HAMS was greater than 20% of the diet, whereas butyrate was increased with 10% of HAMS in the diet and further increased with 40% compared to the diet with only digestible corn starch. On the other hand, faecal acetate and total SCFA increased with 10, 20, and 40% of HAMS in the diet, but no difference was observed for propionate and butyrate. A significant correlation was found between major SCFA and caecal pH. Another interesting indirect effect observed [33] is that colonic DNA damage decreased with the increasing HAMS content of the diet. This effect was positively correlated with the major SCFA and to the colonic pH. The strongest inverse correlation was observed between caecal butyrate and the level of DNA damage. In addition, a strong negative correlation was also observed between the mucus layer thickness and colonic DNA damage.

Due to the ability of the IM to rapidly alter its physiology, it is able to adapt to different dietary carbohydrates from meal to meal. Few studies considered in the systematic review have evaluated the effect of digestible carbohydrates on IM metabolism and none have provided information on microbial composition. This could be due to the fact that most of the digestible carbohydrates are metabolized and digested in the upper gut and do not reach the colon. However, it is worth noting that a large intervention study, found during a subsequent hand search, has reported that high carbohydrate diets increase saccharolytic bacteria including bacteroides and bifidobacteria, which have been independently associated with improved body energy regulation [44].

### Data from a human study on dietary proteins

The change in IM composition and activity in healthy volunteers were investigated when the usual diet was shifted to an animal- or a vegetable-based diet [38]. The animal-based diet had increased fat and protein and decreased fibre content, whereas the vegetable-based diet had increased fibre and decreased fat and protein contents. The authors found differences in the metabolite production, where the vegetable-based diet led to an increase in faecal acetate and butyrate content. On the other hand, the animal-based diet led to an increased production of isovalerate and isobutyrate. The animal-based diet significantly increased the levels of faecal deoxycholic acid (DCA) which is a secondary metabolite of bile acids produced by microbial metabolism. Elevated DCA concentrations may have contributed to microbial disturbances in the animal-based diet, as this bile acid can inhibit the growth of members of the Bacteroidetes and Firmicutes phyla. Changes in IM composition due to the shift from the animal- to the vegetable-based diet were also investigated through 16S rRNA Illumina sequencing [38]. There was a significant increase in diet-associated-diversity that was unique to the animal-based diet. This effect was reversed 2 days after this diet ended. The animal-based diet increased abundance of *Alistipes, Bilophila* and *Bacteroides,* which is consistent with the higher bile acid secretion induced by the higher fat intake. It also decreased the levels of Firmicutes such as *Roseburia*, *E. rectale*, and *R. bromii* that metabolize dietary plant polysaccharides. The animal-based diet was also associated with increased expression of bacterial genes for vitamin biosynthesis, degradation of polycyclic aromatic hydrocarbons, and increased expression of β-lactamase genes. There was a significant positive correlation between subjects’ fibre intake over the past year and baseline intestinal *Prevotella* levels. Significant positive relationships between clusters composed of putrefactive microbes (i.e., *Alistipes putredinis* and *Bacteroides* spp.) and isovalerate and isobutyrate, which are end-products of amino acid fermentation, were also found [38].

### Data from animal studies on dietary proteins

In relation to the absorption of proteins in the gut [45], a meta-analysis of the results of studies performed in pigs observed that the apparent ileal and total tract digestibility of crude proteins decreased by 15–20 and 10%, respectively, when β-glucans increased up to 6% of the diet. The authors also observed an effect with xylose which was lower than β-glucans. Others have studied protein recycling and the contribution of microbial lysine to amino acid metabolism in growing rabbits [37]. Animals were divided into three groups: one group received a control diet, one group received a diet supplemented with the isotope ^15^NH_4_Cl, and one group initially received the control diet, and then, the diet supplemented with the isotope while wearing a neck collar to avoid caecotrophy for the last 10 days of the test period which lasted 30 days in total. They observed differences in the pattern of amino acids between caecal bacteria and caecotrophic material, with tyrosine and methionine being higher and threonine and proline lower in the caecal bacteria compared to the caecotrophic material. In the neck collar group, lysine of caecotrophes showed lower ^15^N enrichment compared with bacteria in the isotope group, due to dilution with the endogenous lysine of the mucous envelope. As for lysine, enrichment in most amino acids, except proline, was higher in bacteria than in caecotrophes. The contribution of microbial lysine to total absorption represented about one-quarter of the total absorbed lysine, mostly due to the caecotrophy process (97%) and only 3% to direct intestinal absorption. Others have examined the effect of a high-protein diet with starch in Beagle dogs [34] and found a higher faecal pH in the high-protein group compared to the control group. Total SCFA, particularly acetate and propionate, were significantly lower in the high-protein diet group compared to the control and the high-starch diet groups. Valerate and BCFA were higher following the high-protein diet compared to the control and the high-starch diets. The effect of the source of dietary proteins in rats on metabolic effects has been evaluated [36].

The effect of the source of dietary proteins has also been evaluated in rats fed diets providing 20% protein from soy, casein, or fish for 16 weeks. While no differences were observed in caecal pH, some differences were observed in caecal SCFA. The fish protein diet led to an increase in total SCFA and lactic acid, which was two times higher than in animals fed the casein or the soy protein diets. Butyrate concentration was greater after the soy diet compared to the two other groups. No differences were observed for acetate and propionate. Caecal indole concentration was two times higher with the fish and soy diets compared to the casein diet. H_2_S and phenol were also higher with soy and fish diets versus the casein diet. Ammonia tended to be higher with the fish diet compared to the other diets.

The effect of two diets, one with medium protein and carbohydrates (MPMC) and one with high protein and low carbohydrates (HPLC), was investigated on the IM structure in kittens [39]. Deep shot-gun sequencing revealed higher species richness and diversity was induced by the HPLC diet compared to the MPMC diet. Moreover, there was higher proportion of rare genera in the faecal microbiota of kittens fed the HPLC compared to the MPMC diet. Interestingly, the IM compositions were more similar within the HPLC group compared to MPMC group. The microbiomes of kittens fed the MPMC diet included bacteria with genes with a greater abundance in pathways associated with the biosynthesis of amino acids, vitamins, fatty acids, and peptidoglycans; the glycolytic, tricarboxylic acid cycle, and pentose phosphate pathways; and oxidative phosphorylation and the metabolism of purines, pyrimidines, and sugars. In contrast, the microbiomes of kittens fed the HPLC diet had greater abundance of genes associated with flagellar assembly, chemotaxis, sporulation, and two-component systems. *Fusobacterium, Clostridium, Eubacterium, Ruminococcus, Bacteroides, Desulfovibrio,* and *Proteobacteria* were significantly more prevalent in HPLC group compared to the MPMC group. *Fusobacterium, Clostridium,* and *Desulfovibrio* are sometimes associated with intestinal disease, but the kittens in this study were healthy. Therefore, their presence was more likely due to their ability to ferment protein-based substrates. On the other hand, there were more *Firmicutes* and *Actinobacteria* in MPMC group compared to HPLC group. *Megasphaera* were in much greater proportions in kittens fed MPMC compared to HPLC. *Megasphera* have the ability to ferment lactate into several SCFA, including butyrate. In the same way, *Bifidobacterium* and *Acidaminococcus* were predominant genera and in much greater abundance in the MPMC- versus HPLC-fed kittens as well as *Selenomonas* and *Prevotella*. *Selenomonas* have the ability to use a wide variety of nitrogen- and carbon-based substrates. These results confirm data obtained in humans, where it was observed that *Bacteroides* tend to be more abundant in those eating diets high in protein and animal fats and *Prevotella* in those eating high carbohydrate diets [46]. The more diverse and less variable composition of IM observed for the HPLC diet reflects a specialized microbiota, while the less diverse and more variable structure on the MPMC diet reflects a more generalised microbiota.

The type of protein also has an impact on the composition of the IM [36]. 16S rRNA gene-denaturing gradient gel electrophoresis (DGGE) and pyrosequencing were used to show that there was greater bacterial diversity with a soy diet and less diversity with a casein diet. Lactobacilli were detected in all groups but not of the same strain. The most typical bacteria with the casein diet were *B. pseudolongum,* while it was Bacteroidetes in the soy diet group. Moreover, 41% of the DGGE bands were identified as *C. bifermentans, C. orbiscindens, C. xylanolyticum,* or other Clostridiales. Lachnospiraceae and *P. excrementihominis* were detected only with fish diet. There was no difference at the phylum level between the three groups with Firmicute*s* predominating and the rest being Bacteroidetes. There was more Ruminococcaceae with the soy diet than with the two other groups. With this detection method, there were more lactobacilli with the fish diet followed by the casein diet and the soy diet displaying the lowest amount of lactobacilli. Bacteroidaceae were only detected with fish diet, whereas with the pyrosequencing method, Lachnospiraceae were dominant in casein and soy diets. *L. incertae* Sedis and *Enterococcus* were detected only following the soy diet.

In summary, while only a small number of recent studies complied with the review criteria, high-protein diets generally led to a decrease in the total SCFA production and in particular of acetate and propionate. On the contrary, valerate and BCFA concentrations, especially isovalerate and isobutyrate, were increased. Some studies have also shown increased concentrations of secondary metabolites of bile acids, particularly of DCA and of ammonia. Considering the effect of protein on IM composition, it was interesting to see a greater diversity in the microbes present in the gut and higher proportion of rarer genera. In humans, there was an increase in *Bilophila, Alistipes*, *Bacteroides*, and several bacteria from the Firmicutes phylum (*Roseburia*, *E. rectale*, and *R. bromii*). The increases in *Alistipes* and *Bacteroides* were correlated with the higher concentrations of BCFA. In animal models, increased concentrations of *Fusobacterium, Clostridium, Eubacterium, Ruminococcus, Bacteroides, Desulfovibrio,* and Proteobacteria were observed, whereas Firmicutes, Actinobacteria, *Megasphaera*, *Bifidobacterium, Acidaminococcus, Selenomonas,* and *Prevotella* were more prevalent in the medium protein diet compared to the high-protein diet. These results are not totally consistent with the observations in humans possibly due to the differences in the composition of the IM in these different models. Moreover, different methods are used to characterize the IM composition, leading to the observation of different results, highlighting the difficulty in comparing and interpreting results across studies utilizing different methodologies.

### Data from animal and human studies on dietary lipid

Results from a study in which rats fed for 20 weeks with a control diet or a high-fat diet with or without *Agaricus blazei* Murill extracts showed that the high-fat diet led to a slight but significant decrease in total bacterial count. There was also a profound decrease (by about 100-fold) in *Lactobacillus* spp. No effect was observed on *Bifidobacterium* spp. [21]. Moreover, differences were in lipid metabolism were observed when two strains of mice (129S6 and BALBc) were fed with diets providing either 5 or 40% of fat (consisting of 40% lard and 8% corn oil) for a 4 month period [41]. In striking contrast to the BALBc mice, the 129S6 mice on the high-fat diet mainly excreted methylamines, namely, dimethylamine, trimethylamine (TMA), and trimethylamine oxide (TMAO), which are choline derivatives produced by the IM. No evaluation of the IM was performed. It has been shown that these metabolites are solely derived from commensal bacteria, and not from mammalian, metabolism. Indeed, GF mice do not excrete TMA and thus indicate the fundamental role of the IM in TMA production from its precursor choline. Foods rich in phosphatidylcholine, which predominantly include eggs, milk, liver, red meat, poultry, shell fish, and fish, are believed to be the major dietary sources for choline, and hence TMAO production. Two other studies [40, 42] have investigated the effect of IM and dietary fat on the production of methylamines. Free-living lacto-ovo-vegetarian women were fed six n-3-enriched eggs per week or six non-enriched eggs per week or an egg-free control period for 8 weeks. The n-3 enriched egg treatment led to higher plasma free choline and betaine versus the egg-free period. No difference was observed between the enriched and non-enriched egg-treatment groups, even with regard to plasma TMAO concentrations [40]. In a challenge test [42], the conventional and GF mice were fed egg-yolk with previous or no administration of antibiotics. TMAO was detected in the control mouse group only; the GF mice or mice having received antibiotic treatment had no or very low concentrations of TMAO.

Few data are available to allow conclusions to be drawn regarding the impact of the IM on dietary fat. Most of the studies retrieved were dealing with the production of methylamines. Indeed, high-fat diets led to increased production of TMA and TMAO, which are risk factors for cardiovascular disease and TMAO is considered both a biomarker and a renal toxin [47]. Concerning the IM composition, high-fat diets appear to lead to reduced diversity with a report of a decrease in *Lactobacillus* spp. [21].

### Impact of the intestinal microbiota on the metabolism of fibre

Recent definitions of fibre have included polysaccharides, such as inulin, and oligosaccharides, such as FOS and galacto-oligosaccharides (GOS), as sources of dietary fibre based on their physiological effects. Due to the wide variety of dietary fibres with different structures and chemical compositions, distinct differences in fermentation patterns are observed. Fibre and prebiotic oligosaccharides are known to resist digestion in the small intestine and reach the colon, where they are fermented by the IM. A total of 23 publications were identified. Of these, seven consisted of human trials [10, 16, 48–52], and 16 studies were conducted using animal models [26, 53–67]. The number of subjects in the human trials varied from 10 to 58 and the study duration varied from 2 to 12 weeks.

### Data from human studies on fibre

A randomized controlled trial [51], including 30 obese women consuming a mixture of inulin and oligofructose (16 g/day) or placebo, was conducted for 12 weeks. IM changes after dietary intervention were monitored using phylogenetic microarray and qPCR analysis of 16S rDNA. Significant increases were found for the Firmicutes groups *Clostridium* cluster IV and XIVa. At genus and species levels, significant increases were found for bifidobacteria, lactobacilli, and *F. prausnitzii* and decreases in *Propionibacterium, B. intestinalis,* and *B. vulgatus* were observed. In addition, the prebiotic treatment significantly decreased post-oral glucose tolerance test glycaemia. Interestingly, several correlations were found between changes in the faecal microbiota and host metabolism. Levels of faecal *Bifidobacterium* spp. and *F. prausnitzii* correlated negatively with serum lipopolysaccharide levels. In contrast, *Propionibacterium* and *B. vulgatus* were both decreased by the inulin-type fructans, and positively correlated with plasma levels of phosphatidylcholine and lactate, which were associated with lower adiposity. Moreover, in this human study, changes in *Propionibacterium* and *B. vulgatus* positively correlated with changes in fat mass and glucose homeostasis. In addition, significantly increased levels of *Collinsella* were found with inulin-type fructan consumption, and these correlated with higher urinary levels of hippurate, a gut-derived metabolite which is decreased in diabetes and obesity.

A mixture of pea fibre and FOS supplemented at 14 g/l of formula provided to 10 healthy adults for 2 weeks induced a significant increase in faecal bifidobacteria in comparison with a fibre-free diet as measured by fluorescence in situ hybridization (FISH). Both the fibre-supplemented and fibre-free diets were shown to decrease the abundance of faecal *F. prausnitzii* and *Roseburia* spp. compared with baseline. The abundance of the *Bacteroides* group decreased significantly during the fibre-fee diet. Faecal SCFA levels decreased significantly on the fibre-free diet, while only butyrate was reduced following the fibre-supplemented diet. As highlighted earlier, a significant positive correlation was found between the change in proportion of *F. prausnitzii* and acetate and butyrate following the fibre-supplemented diet and between the change in proportion of *F. prausnitzii* and butyrate following the fibre-free diet [10]. Two randomized controlled trials in healthy adults demonstrated that supplementation with wheat bran extract, a fraction rich in arabinoxylan oligosaccharides, also increases faecal *Bifidobacterium* spp. content relative to control groups [50, 52]. Cloetens et al. [52] investigated 20 healthy adults consuming their usual diet supplemented with 13.9 g/day of wheat bran extract or maltodextrin. In particular, the abundance of *B. adolescentis,* as measured by quantitative PCR, was found to be significantly increased and the levels of lactobacilli were decreased compared with control. In the other study [50], significantly increased levels of bifidobacteria were detected by FISH upon consumption of 10 g/day of wheat bran extract by 58 healthy adults. No significant changes in total bacteria or other bacterial groups were found in the respective studies [50, 52]. In addition, consumption of 10 g/day of wheat bran extract significantly decreased urinary *p*-cresol, a marker of proteolytic fermentation, and significantly increased faecal total SCFA levels and, in turn, decreased stool pH [50]. In two randomised, double-blind, placebo-controlled trials [49], the effect of different dosages of the soluble fibre Nutriose, a resistant dextrin derived from wheat or maize starch, on the faecal microbiota was investigated. Forty-eight healthy individuals received 10, 15, or 20 g of Nutriose per day or a product control (glucose) for 14 days in the first trial. In the second trial, 8 g/day Nutriose or the product control was given for 14 days to 40 healthy individuals. Nutriose, in general, increased the number of *Bacteroides* and decreased *C. perfringens* numbers in faeces. In addition, the β-glucosidase activity was enhanced and faecal pH was lowered by Nutriose consumption. The authors hypothesized that the increased fermentation activity led to a lower colonic pH (although no significant change in SCFA was shown), which may have inhibited the growth of potential pathogens like *C. perfringens*.

The impact of a WG rich diet and a refined-grain (RG) diet has also been examined [16, 48]. Due to the inherently high-fibre content of WG cereals compared with their refined counterparts, it has been hypothesized that WG cereals may play a role in modulating the IM and alter intestinal transit. In a randomized, researcher-blinded, cross-over study with 17 healthy subjects that received a 2-week intervention diet, faecal *C. leptum* and *Enterococcus* spp. populations were shown to increase after the WG diet, along with a trend for decreased faecal water pH and increased stool frequency compared with the RG diet. The *C. leptum* population is known to contain a number of bacterial species adapted for utilizing fibre as a substrate and producing butyrate. The observed trend for a decrease in faecal pH with the WG diet fits with the increase in butyrate-producing bacteria. On the basis of metabolic changes, as measured in plasma, urine, and faecal water, the WG diet affected a variety of pathways, including protein, lipid, and microbial metabolism. Protein-derived IM metabolites were lower during the WG diet, suggesting that more carbohydrate was available in the distal colon, reducing the use of protein as a substrate. The WG diet lowered faecal excretion of isovalerate, a product of amino acid metabolism by the IM also found to decrease during diets high in fermentable carbohydrates in other studies. The observed changes in IM composition were also reflected in the urinary metabolic profile, e.g., a lower urinary excretion of 4-hydroxyphenylacetate; a colonic metabolite of tyrosine and phenylalanine breakdown (by *C. perfringens* and *C. difficile,* respectively) was evident. The urinary metabolic profiling also revealed differences in response to the diets due to gender differences: urinary fumarate differed between the WG and RG group in woman, but not in men. Interestingly, this gender difference has also been observed in other metabolic profiling studies.

### Data from animal studies on fibre

Rats provided a diet containing either unprocessed pomace from apple juice pressings (61% fibre), or ethanol- and ethanol/acetone-extracted pomace (66% fibre) for 4 weeks [64] exhibited an increase in caecal SCFA and a decrease in BCFA accompanied by lowered small intestine and colonic pH compared to control-fed rats. Jeon and Choi [62] fed rats with a normal diet, a diet containing 10% lactic acid bacteria and a diet with 2% lactic acid bacteria-fermented germinated barley for 6 weeks. Germinated barley contains glutamine-rich proteins and insoluble dietary fibres and this fibre supplementation resulted in enhanced caecal SCFA production. The primary outcome of the study was the improvement of intestinal transit and an increase of faecal mass in a loperamide-induced model of constipation.

The faecal metabolic changes in different IM models were monitored when a probiotic or a symbiotic (probiotic plus prebiotic fibre) was supplemented to the diet [65]. GF mice were colonised either by exposure to normal environment (conventionalised) or with a simplified human baby microbiota (HBM), and the conventional mice served as controls. Interestingly, HBM mice displayed different metabolic patterns in the faeces in comparison with the conventional and conventionalised mice. The level of acetate, butyrate, and propionate was decreased, but also lactate, 5-aminovalerate, bile acids, tyrosine, glucose, and U3 (unassigned metabolite, most likely fatty acid related compound) amounts were lower, while choline, citrate, creatine, and succinate levels were increased. In addition, between the conventional and conventionalised mice, some differences in the content of fatty acids in faeces were observed. This has also been found in other ex-GF mice previously and might be a long-term consequence of the GF state. The conventionalised mice only differed from conventional mice in a lower intestinal lactobacilli count. The IM of the HBM mice harboured higher populations of enterobacteria, bacteroides, and staphylococci. *C. perfringens* was present and lactobacilli were absent in comparison with the conventional and conventionalised mice. Furthermore, when HBM mice received a probiotic diet with or without GOS (3 g/100 g) for a period of 14 days, the supplementation with the GOS resulted in higher numbers of bifidobacteria and lactobacilli, lower levels of unassigned fatty acids and higher content of oligosaccharides, and a higher increase in acetate over time [65]. Others have studied the impact of different types of GOS on the IM [60]. For 14 days, rats were fed a standard diet alone or a standard diet with either GOS derived from lactulose (GOS-Lu) or a GOS derived from lactose (GOS-La). Faecal samples revealed an increase in bifidobacteria with both GOS types. Compared to each other, GOS-Lu had a significantly stronger bifidogenic effect, whereas GOS-La increased lactobacilli more significantly. Compared to controls, both types of GOS raised counts of *E. rectale* and *C. coccoides* in the faecal microbiota. In relation with GOS metabolism, the caecum and faecal samples clearly showed that both GOS were readily fermented in the large intestine. In addition, a 3-week dietary enrichment study with GOS resulted in a reduced energy intake of GOS-fed rats [26] and increased colonic lactic acid levels, a primary GOS metabolite from bifidobacteria and lactobacilli species that becomes abundant on diets containing GOS [26]. This reduction in energy intake in response to GOS diets is in line with results reported for other fermentable fibres, e.g., FOS, inulin, and RS. Epididymal fat weight, a marker of abdominal fat mass, was distinctly lower in GOS-fed rats. This could be a general consequence of reduced energy intake and/or a specific, direct effect of GOS on fat metabolism, e.g., increased lipid oxidation.

SCFA are postulated to stimulate secretion of GLP-1 in vitro, which in turn stimulates insulin secretion and inhibits gastric emptying and intestinal transit. However, it is unclear the degree to which this occurs in the colon. It has been suggested that the IM increases GLP-1 levels through production of SCFA [67]. This was confirmed with mono-colonization of genetically modified mice with two representatives of the IM, *E. coli* and *B. thetaiotaomicron*, with resulting lower and higher fermentation abilities, respectively and thus SCFA producing abilities [67]. The role of GLP-1 in decreasing small intestinal transit could be an adaptive response for promoting nutrient absorption in response to insufficient energy availability in the colon. Elevated GLP-1 levels and slower intestinal transit times have also been reported in anorexia nervosa patients [68, 69], suggesting that this function may be conserved in humans. Even though the pre-meal plasma levels of GLP1-1 were less elevated compared to the standard diet, GOS has also been shown to increase (3.5 fold) the colonic mucosal gene expression of the GLP-1 precursor proglucagon [26]. Anatomical and physiological factors might explain this, as a significant proportion of GLP-1 is produced in cells in the proximal parts of the small intestine, which receive minimal exposure to colonic GOS fermentation metabolites.

The effects of porphyran, a water-soluble dietary fibre from algal cell wall, were tested in a mouse model for type 2 diabetes. Mice received a high-fat diet (14% fat), 5% porphyrin, or two kinds of porphyrin salt with 5% cellulose as control [63]. Besides improvements in plasma insulin levels, insulin resistance index, and plasma adiponectin levels, changes in caecal SCFA content and IM composition were measured by gas chromatography and real-time PCR. The content of caecal propionate was significantly higher when porphyrin was fed. SCFA are ligands for the G protein-coupled receptors GPCR41 and GPCR43 and may impact adipocytokine production. Interestingly, the increase of propionate was not significant when the porphyrin salts were fed, suggesting that arginine and potassium might influence the metabolism of the caecal microbiota. In all porphyrin groups, higher numbers of *Bacteroides* spp. and fewer *C. coccoides* were detected in the caecum compared to controls [63].

Neyrinck et al. [66] found that intake of chitin–glucan fibre can partly reverse the impact of a high-fat diet on the IM in animals. Mice received a high-fat diet (60% fat, 20% carbohydrates, and 20% proteins) or a high-fat diet supplemented with 10% fungal chitin–glucan for 28 days. The high fat diet decreased total bacteria number in caecal samples and in particular *Bacteroides/Prevotella* group, *C. coccoides*/*E. rectale* cluster, *Lactobacillus* spp., and *Roseburia* spp. Bifidobacteria were increased and suggest that the effect may depend on the composition of the high-fat diet. With the addition of the chitin–glucan fibre, the high-fat diet induced decrease in the *C. coccoides/E. rectale* cluster and *Roseburia* spp. was partly compensated by a modest increase in number of *Bacteroides/Prevotella* group. In addition, the high-fat induced body weight gain, fat mass development, plasma cholesterol concentration, hepatic triglycerides, and other metabolic markers were also counteracted.

The impact of oligofructose consumption in genetic or diet-induced obese and diabetic mice has been studied [57, 58]. In contrast to the human trial [51], a decrease in the caecal abundance of the Firmicutes, but increase in Bacteroidetes as measured by pyrosequencing was reported [58]. Similar to the human study, at the genus level, significant increases were found for *Bifidobacterium* spp. by quantitative PCR. In both animal trials, caecal *Roseburia* spp. were found to be decreased. The authors concluded that modification of the IM by oligofructose leads to improvement of glucose homeostasis and leptin sensitivity and targets enteroendocrine cell activity in obese diabetic mice. Interestingly, positive associations were found between *Anaerotruncus* and glucose intolerance, intestinal permeability, plasma triglyceride content, and lipid muscle content and between *Clostridium lactifermentas* and glucose intolerance, intestinal permeability, and lipid muscle content, and finally between *Streptococcus intermedius* and intestinal permeability. Others [57] have shown that oligofructose consumption counteracted the increase in GPR43 over-expression induced in the adipose tissue by a high-fat diet. GPR43 is a key factor controlling adipose differentiation leading to the accumulation of large adipocytes upon a high-fat diet. Interestingly, De Vadder et al. [56] identified another mechanism by which FOS can exert their effects. FOS supplementation significantly decreased colonic levels of Firmicutes while increasing levels of Bacteroidetes in mice. These changes correlated with significant increases of propionate in the portal vein which led to activation of intestinal gluconeogenesis and, as such, induced beneficial effects on glucose and energy homeostasis.

Oat bran supplemented to an atherogenic diet for 4 weeks showed a significant increase in the total bacterial abundance and more specifically in the caecal abundance of *B. fragilis* and *Akkermansia* in two sub-strains of C57BL/6J mice in comparison with the controls. In addition, one of the sub-strains showed a significant decrease in plasma cholesterol and plasma fructosamine levels, a significant increase in faecal secretion of bile acids as well as an increase in the hepatic expression of enzymes in the bile acid synthesis pathways CYP7A1 and CYP8B1. However, a correlation between the changes in the microbial community and the cholesterol lowering properties of oat bran was not observed [53]. In another study, blueberry husks were added to a control diet (120 g dietary fibre/kg diet) and fed to rats for 5 days [54]. The rats consuming the diet with blueberry husks gained significantly more weight than the rats consuming the control diet. In addition, the faecal wet weight was significantly increased. Some 39% of the ingested dietary polysaccharides from the blueberry husks were excreted which was suggested to be due to the high lignin content. The caecal counts of lactobacilli, bifidobacteria, and Enterobacteriaceae significantly decreased, while the total SCFA content in the caecum, distal colon, and faeces of the rats was significantly higher for the blueberry husks diet- than the control diet-fed rats. In the caecum, significantly higher levels of acetic acid and l-lactate were found, while propionate and minor acids were significantly decreased. In the distal part of the colon and in the faeces significantly, higher proportions of propionate and butyrate, respectively, were found, with blueberry husks consumption [54]. It has been suggested that the effects may be due to the anti-microbial action of the phenolic compounds in the blubbery husks, in addition to the fibre.

Hydroxypropyl methylcellulose, a synthetic modification of the natural polymer cellulose, supplemented at 10% (w/w) to a high-fat diet for 4 weeks was found to significantly increase the levels of faecal Erysipelotrichaceae and faecal and caecal Peptostreptococcaceae while decreasing the abundance of faecal Lachnospiraceae and faecal and caecal Ruminococcaceae in high-fat diet-fed mice as measured by pyrosequencing. At the genus level, hydroxypropyl methylcellulose led to significant decreases in *Johnsonella* and *Lactobacillus* and significant increases in *E. incertae* Sedis and *Peptostreptococcus*. In addition, metabolic changes were found upon hydroxypropyl methylcellulose consumption, i.e., significantly reduced total cholesterol, high, low, and very low density lipoprotein cholesterol, leptin, liver triglycerides, and liver percentage adiposity. In contrast, the product significantly increased levels of faecal saturated, unsaturated, and trans-saturated fat, monoacylglycerides/free fatty acids, bile acids, and energy. Positive associations between weight changes and faecal saturated fat were found for caecal Erysipelotrichaceae and caecal Erysipelotrichales, respectively. Faecal abundances of Lachnospiraceae were negatively correlated with energy intake and caecal Porphyromonadaceae correlated positively with liver-free cholesterol [55]. The impact of polydextrose supplementation (30 g/day for 3 weeks) on the IM composition and activity, and immunological parameters was investigated in 20 healthy pigs fed a high-energy-density diet [59]. Polydextrose was found to be fermented at an even rate along the intestine and about 66% of the amount present in the distal small intestine was recovered at the distal part of the colon. Levels of faecal SCFA and tryptamine were significantly decreased in the distal colon upon polydextrose supplementation, while spermidine and spermine were significantly increased. A tendency towards a decreased expression of mucosal cyclo-oxygenase-2 was reported. No significant changes in the composition of the IM community as measured by % G + C profiling and FISH were observed [59].

While investigating the effects of eight different dietary fibre containing diets on metabolic risk factors, changes in the formation of SCFA and the caecal microbiota were measured [61]. Either a low-fat diet (50 g fat/kg) or a high fat diet (280 g fat/kg) containing either, no fibre, pectin, guar gum, or a pectin-guar gum mixture was fed to rats for 2 or 6 weeks, respectively. In general, the high-fat diet reduced the butyrate concentration (in blood, serum, and caecal samples) and increased the succinate caecal levels significantly after 2 weeks. Butyrate and succinate were generally higher in all groups that received fibres in comparison with the groups on the fibre-free diet. The amount of total SCFA in blood serum and the caecum was lowered by diets without fibre, with pectin or with guar gum reduced, but not with the mixture of fibres. This has also been shown in the previous studies, suggesting that this is due to a change in the IM. Among the high-fat groups, all fibre interventions led to a higher serum content of acetate in comparison with the fibre-free intervention. Interestingly, guar gum and the mixture of fibres counteracted the effect of the high-fat induced decrease of butyrate. In addition, the IM composition analysed from caecal samples changed considerably in response to each dietary fibre diet. Rats fed guar gum showed higher numbers of *Bacteroides*, which relates to butyrate levels in the serum and caecum as well as caecal propionate levels. The fibre-free diet led to an increase of *Akkermansia* in the caecum. For the pectin group, high individual variation was detected and no distinct IM changes could be identified, but an increased formation of acetate was noted. In addition, in this study, it was also shown that dietary fibres reduced weight gain, lowered the amount of liver fat, hepatic cholesterol, and triglycerides, and decreased inflammatory markers. Pectin and the mixture of fibres seemed to have the strongest impact [61].

### Impact of the intestinal microbiota on the metabolism of resistant starch

While the majority of dietary starch ingested is completely digested, a small proportion, known as RS, escapes digestion in the small intestine and reaches the colon. RS is classified into four types of starch, physically inaccessible forms, and certain granular forms that are resistant to enzyme digestion, retrograded, and chemically modified starch. RS provides the colonic bacteria with the largest single-source of diet-derived energy. Seven publications relating to RS were identified. Of these, two reported studies in humans [70, 71] and five in animals [24, 32, 72–74]. The number of human subjects per study varied from 14 to 46 and study duration varied from 3 to 4 weeks.

### Data from human studies on resistant starch

A randomized cross-over trial including 46 healthy adults [70], showed different responses of the microbial communities to a 4-week diet high in non-starch polysaccharide (NSP in the form of ‘added wheat fibre’) (25 g total fibre and 1 g RS) or high in NSP and RS (25 g total fibre and 22 g RS). A significant increase in the abundance of faecal *R. bromii* was observed in the case of the NSP plus RS diet. In addition, faecal total SCFA, acetate, and butyrate levels were significantly increased upon consumption of the NSP plus RS diets and the stool pH significantly decreased. The impact of diets rich in RS or NSP on the IM in an obese population has also been investigated [71]. Fourteen obese men with metabolic syndrome participated in a 10-week intervention trial consuming four different diets: a run-in maintenance diet for 1 week; a crossover of two diets each for 3 weeks, one diet high in RS type-3 and one diet high in NSP; and finally, a weight-loss diet with high protein and medium carbohydrate levels. Human Intestinal Tract Chip analyses of the faecal microbial communities showed a subjectwise clustering indicating a strong individuality of the microbial community structure and relatively limited effects of the diets. Overall, the impact of the diet explained about 10% of the total variance in IM composition. However, comparison of the IM composition on an individual basis did show significant differences between the diets. RS was found to stimulate mainly bacteria belonging to *Clostridium* cluster IV such as *O. guillermondii* and *R. bromii,* while bacteria belonging to *Clostridium* cluster XIVa decreased. The NSP diet was found to stimulate the growth of bacteria belonging to the family Lachnospiraceae. Faecal SCFA were found to be significantly lower on the RS and weight-loss diet in comparison with the NSP and maintenance diets. Interestingly, propionate production was positively correlated with faecal Bacteroidetes abundance, and in addition, a positive correlation was found between faecal bifidobacteria and plasma insulin. Remarkably, the responsiveness of an individual’s IM to a dietary intervention, either RS or NSP or weight loss, was found to be dependent on the initial microbial diversity of the individual. Those with a low initial microbial diversity were identified as so-called ‘responders’ versus individuals with a high initial microbial diversity or so-called ‘non-responders’. The authors indicated that this result was consistent with the concept that phylogenetic diversity promotes ecosystem stability.

### Data from animal studies on resistant starch

Further insights into the impact of RS consumption on the IM and host metabolism have been generated in animal trials. Rats fed potato-RS diets with casein or cooked beef showed increased abundance of *Bifidobacterium* in the caecum and both *Bifidobacterium* and *Lactobacillus* in the colon as revealed by quantitative PCR [24]. A significant increase in acetate was found in comparison with a cellulose diet. No decrease in markers of proteolytic fermentation, phenol, and *p*-cresol was found, although longer colon crypts and a greater number of goblet cells per crypt were found upon RS feeding indicating improved colonic health. In pregnant rats fed RS type 2 (high-amylose maize starch), an improvement of insulin sensitivity and glycaemic control was shown in comparison with rats fed an equal energy dense control diet [32]. In addition, pups born to rats on the RS diet had lower fasting glucose levels. Caecal and faecal pH were found to be significantly decreased, caecal SCFA levels significantly increased, and *Bacteroides, Bifidobacterium, Lactobacillus,* and *Clostridium* cluster IV populations significantly increased in the RS-fed adult rats after weaning. In addition, β-cell relative densities were significantly decreased and serum total GLP-1 levels significantly increased. The latter was suggested to result from the increased production of butyrate and linked to the mechanism of improved glycaemic control. Similarly, significantly higher caecal propionate and plasma GLP-1 levels as well as significantly decreased liver triacylglycerides were reported upon consumption of RS type-2 or hydroxypropylated RS type-2 in rats [73]. According to the human study results, an increase in faecal *R. bromii* and propionate levels in the distal colonic digesta was found in azoxymethane-treated rats (a model for colorectal cancer) consuming a diet supplemented with RS type-2 for 31 weeks. Consumption of butylated RS type-2 was also shown to increase propionate and butyrate levels in the distal colonic digest and faecal levels of *L. gasseri* and *P. distasonis* [72]. In the same model, Le Leu et al. [74] showed a significant increase in the caecal and colonic total SCFA levels, as well as propionate and butyrate levels upon RS type-2 consumption. A non-significant trend towards protection against colorectal cancer was observed.

In summary, few recent studies have investigated the impact of specific IM clusters on the metabolism of various fibres or RS. Most intervention studies deal with the question of how fibre and RS change the IM composition. Such results give rather indirect information on which IM might be involved in the degradation processes. More studies are needed to clarify which IM composition results in an improved availability of various fibres and RS for the host. In addition, monitoring of the production of metabolites in a more precise manner is required to obtain better insights into the specific metabolic pathways and microbial species involved. In relation with dietary fibre, studies have associated increased microbial richness with diets high in fibre, vegetables, and fruits. In controlled human studies, consistent data exist that show a correlation between the intake of fibre and an increase in bifidobacteria and SCFA, in particular butyrate. This leads to lower pH values in the intestinal tract, which is considered beneficial. Regarding changes in the composition of the faecal microbiota, there are conflicting data or data that require more detailed examination of the bacteria at species level; e.g., in one study, bacteroides in general were increased after fibre supplementation, whereas other studies revealed a decrease in *Bacteroides intestinalis* and *Bacteroides vulgatus*. Overall, fibre intake and its effects on metabolites and IM composition were associated with a positive impact on glucose and fat metabolism in humans.

### Impact of the intestinal microbiota on the metabolism of polyphenols

Polyphenols are a large class of structurally diverse chemicals found in plants that can be separated into three main subclasses, the phenolic acids (derivatives of benzoic acid and cinnamic acid), stilbenoids (stilbenes), and the flavonoids. The flavonoids are further sub-divided into the flavonols, flavones, isoflavones, flavanones, anthocyanidins, and flavanols (flavan-3-ols). The majority of these polyphenols, particularly the flavonoids, occur naturally in their glycosylated form (conjugated to a sugar moiety). In their glycosylated and polymeric forms, these polyphenols are usually poorly absorbed in the upper gut and pass into the large intestine, where they become substrates for the IM. Glycosides can be cleaved to liberate the aglycone and complex structures can be broken down into their monomeric forms which improve bioavailability and alter bioactivity. Although host intestinal mucosal enzymes can hydroxylate some glycosides, the majority are metabolized by enzymes derived from the IM. In vivo studies evaluating the influence of the IM on polyphenol metabolism, and subsequent bioavailability, in both animals and humans are summarized below. The screening approach used identified three human [75–77] and five animal [78–82] studies that met the selection criteria for assessing the interaction between the IM and polyphenols excluding isoflavones. Several studies have characterized the microbial biotransformation of polyphenols using in vitro culture systems. However, the focus for this review was specifically on in vivo studies. The number of subjects in the human studies varied from two to 35 and the study duration varied from single dose to 17 day follow-up.

### Data from human studies on polyphenols

The IM is known to metabolize polyphenols into smaller metabolites through a range of biotransformational strategies including dehydroxylation, deglycosylation, and demethylation. In a human study [76], the microbial metabolism of almond skin polyphenols (884 mg total polyphenols/dose) was characterized in urine and blood using a liquid chromatography–mass spectrometry (LC–MS) approach. Despite the limited sample size of this study (*n* = 2), several microbial metabolites were detected including hydroxyphenylvalerolactones, 5-(dihydroxyphenyl)-γ-valerolactone, and 5-(hydroxymethoxyphenyl)-γ-valerolactone. These compounds arise from the microbial degradation of flavanols and were detected in their glucuronidated and sulfated forms. Through fission of the valerolactone ring, the IM can further metabolize these products to the hydroxyphenylvaleric acids. These compounds are subsequently broken down via bacterial β-oxidation to produce hydroxyphenylpropionic acids (3,4-dihydroxyphenylpropionic acid and 3-hydroxyphenylpropionic acid) and hydroxybenzoic acids (3-hydroxybenzoic acid, 4-hydroxybenzoic acid, protocatechuic acid, and vanillic acid). Additional processing of hydroxyphenylpropionic acids can occur through α-oxidation to hydroxyphenylacetic acids (3,4-dihydroxyphenylacetic acid, 3-hydroxyphenylacetic acid, 4-hydroxy-3-methoxyphenylacetic acid, and phenylacetic acid). All of these microbial metabolites were observed to increase in the urine post-almond polyphenol intake [76]. In addition, 4-hydroxyhippurate, *m*- and *p*-coumaric acids, ferulic acid, and 4-hydroxy-3-methoxyphenylacetic acid were measured in the urine and plasma post polyphenol intake. 4-Hydroxyhippuric acid is formed from the hepatic conjugation of 4-hydroxybenzoic acid with glycine. The hydroxycinnamic acids, *m*- and *p*-coumaric acid, are formed from the dehydrogenation of 3- and 4-hydroxypropionic acids, respectively, and further para-hydroxylation and methylation of *m*-coumaric acid results in ferulic acid. Methylation of protocatechuic acid and 3, 4-dihydroxyphenylacetic acid produces vanillic acid and 4-hydroxy-3-methoxyphenylacetic acid, respectively. Collectively, these findings demonstrate the importance of IM metabolism of polymerized polyphenols and the diverse biotransformation possibilities encoded in the microbiome and their resultant metabolic outputs.

The IM metabolism of flavanols with different degrees of polymerization was studied in six healthy men using a randomized cross-over study design [77]. These flavanols included epicatechin (monomer), procyanidin B1 (dimer), and a purified polymeric procyanidin fraction isolated from cocoa. Both gas chromatography (GC) and LC–MS platforms were used to measure metabolites in urine, blood, and faeces. In these individuals, the IM metabolized the monomeric and dimeric flavanols to products that are absorbed from the intestinal tract. These metabolites were 5-(3,4-dihydroxyphenyl)-valerolactone (DHPV) and 4-hydroxy-5-(3,4-dihydroxyphenyl)-valeric acid (4H-DHPVA). *E. lenta* has been shown in vitro to perform the reductive cleavage of (−)-epicatechin and (+)-catechin to 1-(3,4-dihydroxyphenyl)-3-(2,4,6-trihydroxyphenyl)propan-2-ol, which can then be metabolized by *Flavonifractor plautii* to DHPV and 4H-DPVA [98]. High plasma and urinary excretion of DHPV following flavanol ingestion indicates that a large amount of epicatechin and the vast majority of procyanidin B1 is metabolized by the IM, with plasma concentrations peaking 8 h post-intervention. This indicates that flavanols are absorbed in either their monomeric form or the microbial breakdown product DHPV. In contrast, the same trends were not observed with the larger polymers contained in the cocoa-derived procyanidin extracts [77]. This implies that the IM cannot metabolize these complex flavanols with the same efficiency as the simpler molecules. Indeed, the degree of polymerization appears to be inversely associated with the production of DHPV. The large structure of these compounds is likely to impede uptake into the bacterial cell restricting their breakdown. A high degree of variation was noted across individuals in the formation of DHPV mostly likely attributable to functional variation in the IM across participants.

A metabolomic approach was applied to assess the potential for polyphenols to counteract inflammation and oxidative stress that can occur with intense exercise [75]. Water-soluble polyphenols from blueberry and green tea extracts were provided to long-distance runners (*n* = 35) before intense exercise and alterations in the metabolic composition of their blood was evaluated. This supplement was rich in phenolic compounds (2136 mg gallic acid equivalents) including flavanols, such as anthocyanins and catechins, several hydroxycinnamic acids, and flavonol glycosides of quercetin, kaempferol, and myricetin. While the supplement was not found to alter inflammation or oxidative stress post-exercise, a range of metabolites associated with the bacterial breakdown of phenolic compounds were increased post-exercise including hippurate, 4-hydroxyhippurate, 4-methylcatechol sulfate, and cinnamoylglycine. Circulating arabinose, a pentose sugar associated with blueberry anthocyanins, was also increased in these individuals arising from the bacterial degradation of these flavanols. Interestingly, exercise increased intestinal permeability in these individuals, increasing host exposure to these IM-derived metabolites. As with the previous study investigating flavanol metabolism [77], a large amount of variation was also observed in the circulating amounts of these microbial metabolites across the participants, again most likely reflecting functional variation in the IM. Although the microbial breakdown products of these dietary polyphenols are considered to have lower antioxidant and anti-inflammatory properties than their parent compounds, the enhanced bioavailability of these derivatives may compensate for this reduction in bioactivity [99].

### Data from animal studies on polyphenols

The participation of the IM in the metabolism and bioavailability of polyphenols was also demonstrated in several rodent studies. Baicalin is a flavone found in *Scutellaria baicalensis* Georgi that is used in traditional herbal medicine. It is a biologically active compound that is hydrolysed by bacterial β-glucuronidases in the intestinal tract to the aglycone, and more readily absorbable form, baicalein [100]. Once absorbed baicalein is glucuronidated, in both the liver and intestine, back to baicalin (baicalein-7-glucuronide) and other glucuronide conjugates such as baicalein-6-glucuronide. The importance of the IM in baicalin metabolism was demonstrated by the significant effect on the pharmacokinetic profile of orally administered baicalin observed in rats after antibiotic pre-treatment [79]. The persistence of baicalin in the intestinal contents of rats receiving the antibiotics highlighted the inability of the host to perform this function. This demonstrates the crucial role of the IM in determining the pharmacokinetics of this flavonoid and the extension of the metabolic functionality of the host beyond that encoded within the genome. The bioavailability of all flavonoids, however, was not influenced by the IM. Others [80] have found that the IM had minimal influence on the absorption of the flavones luteolin and apigenin. Here, rats were provided with a *Chrysanthemum morifolium* Ramat extract (200 mg/kg) containing luteolin and apigenin in their glycosylated forms. Flavone deglycosylation was high in the large intestine with a good recovery (70%) of luteolin and apigenin in the caecum and colon of the rats. However, a significant amount of flavone deglycosylation occurred in the rat stomach and small intestine resulting in rapid absorption. As such, the contribution of the IM towards the bioavailability of these flavonoids was negligible. This was confirmed in subsequent experiments administering either antibiotics or emptying the luminal contents of the intestine to minimize the metabolic contribution of the IM. The formation of aglycones was not significantly reduced in these experiments indicating that intestinal glycosidases were the primary contributors to this action. This is consistent with an earlier study that measured the metabolism of luteolin, apigenin, and their conjugates in GF rats [101].

Quercetin glycosides are the most abundant flavonoids in fruits and vegetables and the importance of the IM in their metabolism was demonstrated in the rat [81]. Although host mechanisms exist for the metabolism and uptake of these compounds in the small intestine, their bioavailability is considered to be poor. Consequently, a large amount of flavonoids reach the colonic microbiota which, in turn, hydrolyse the sugar moiety and release the aglycone facilitating the absorption of the flavonoids. However, extensive microbial processing of the quercetin aglycone to 3,4-dihydroxyphenylacetic acid and phloroglucinol can occur reducing its bioavailability and bioactivity [102]. In this study, the authors established that supplementing rats with either the non-digestible disaccharide, di-d-fructose anhydride III (DFAIII), or FOS attenuated the microbial breakdown of the quercetin aglycone. Decreasing this breakdown promoted quercetin availability, increasing systemic levels of quercetin derivatives and their urinary excretion. Faecal excretion of quercetin metabolites, mainly methylquercetin, was also increased in animals provided with DFAIII and FOS. In total, the urinary and faecal excretion of the aglycone was 30% that of the ingested amount in rats fed FOS or DFAIII. In the control animals, a total of 16.4% of the aglycone was excreted, suggesting that almost 85% of the quercetin aglycone was metabolized by the IM. Both DFAIII and FOS serve as fermentable substrates for the IM and these findings show that the provision of alternative microbial substrates can spare dietary components from microbial breakdown increasing their availability for uptake.

The antioxidant properties of polyphenols are suggested to prevent or reduce oxidative stress and, therefore, damage, associated with ischemia–reperfusion (I/R) injury. A mouse study [78] explored the potential for chokeberries and bilberries, rich in anthocyanins to reduce the oxidative stress caused by I/R. Anthocyanins are effective anti-oxidants and are metabolized by the colonic microbiota to absorbable derivatives of phenylpropionic, phenylacetic, and benzoic acids of various different hydroxylation patterns. Following intake of the polyphenol-rich diet, various microbial degradation products of the polyphenols were detected in the caecum and colon of the mice. In the bilberry-supplemented mice, 3,4-dihydroxyphenylacetic acid, a gut bacterial metabolite of proanthocyanidin and quercetin (both known to be present in bilberries), was observed in the large intestine. 3-Hydroxyphenylpropionic acid was also found in the caecum and colon of these animals. This metabolite is considered to derive from the bacterial action on epicatechin, catechin, procyanidins, and chlorogenic acid. In the chokeberry-fed mice, dehydroxylation products of 3,4-dihydroxyphenylacetic acid (3- and 4-hydroxyphenylacetic acid) were measured in the caecum and colon. Many of these aromatic acid metabolites generated by the IM possess antioxidant properties and as they are more readily absorbed than their parent compounds, these biotransformations are considered important in relation with the protective effect of polyphenols, and polyphenol-rich foods, against oxidative stress. Interestingly, bilberries, but not chokeberries, were found to reduce tissue injury associated with I/R. Chokeberries contain a greater amount of highly polymerized procyanidins and this may underlie the reduced antioxidant properties and effectiveness of chokeberries compared to bilberries as the greater degree of polymerization restricts bacterial access for degradation. This is consistent with the observations for flavanols in humans [77]. Another contributing factor may be the greater diversity and complexity of the anthocyanins in the bilberries that survive transit into the colon compared to chokeberries.

Given the therapeutic interest in grape seed polyphenol extract (GSPE) in Alzheimer’s disease, the role of the IM in shaping their bioavailability and bioactivity was determined in rats [82]. GSPE is rich in simple and complex phenolic compounds and the IM are known to metabolize these to more readily absorbable phenolic acids. Following oral administration of GSPE for 11 days, the bioavailability of 12 phenolic acids, produced from the bacterial metabolism of anthocyanidins, were measured by high-pressure LC–MS. These compounds included ferulic acid, hippurate, 3- and 4-hydroxybenzoic acid, 3- and 4-hydroxyhippurate, 3-hydroxyphenylacetic acid, 3-(3,4-dihydroxyphenyl)acetic acid, 3-hydroxyphenylpropionic acid, 3-(3,4-dihydroxyphenyl)propionic acid, 5-(4-hydroxyphenyl)valeric acid, and phenylacetic acid. GSPE intake was found to increase the accumulation of all 12 phenolic acids measured in the caecum, colon, urine, and/or blood. Of these, two phenolic acids (3-hydroxybenzoic acid and 3-(3-hydroxyphenyl) propionic acid) were found to accumulate in the brain at micromolar concentrations. The metabolites were shown to potently disrupt the formation of neurotoxic β-amyloid aggregates that have been implicated in Alzheimer’s disease pathogenesis. Thus, highlighting the potential role of IM-derived microbial metabolites of polyphenols in brain physiology and health maintenance.

Overall, these in vivo studies demonstrate the strong influence of the IM on the bioavailability and bioactivity of a range of dietary polyphenols and their subsequent impact on the host. The extent of polyphenol biotransformation is largely dependent on structure and the degree of polymerization. Presently, there are few in vivo studies exploring genus-level associations with specific polyphenol processing traits with these investigations largely restricted to in vitro studies. Nevertheless, our understanding of the role of the IM in shaping the host visibility of polyphenols and their breakdown products is increasing, as are the methods that can be used to measure this, for example, strategies are evolving, such as prebiotic supplementation to manipulate the microbial interactions to enhance the benefits for the host [103]. Given the growing health benefits associated with polyphenols, optimizing such benefits via the IM is an attractive area of future research.

### Impact of the intestinal microbiota on the metabolism of isoflavones

Isoflavones are naturally occurring plant substances that belong to the ‘phytoestrogen’ class and the effect of their intake is being studied in a range of hormone-dependent conditions. In relation with the metabolism of isoflavones, particularly daidzein, a total of 15 studies were identified that fulfilled the selection criteria. Of these, ten were human studies [83–92] and five were animal studies [93–97]. Number of subjects varied from 11 to 297 and study duration varied from single dose to 20 weeks follow-up.

### Data from human studies on isoflavones

The majority of studies focussed on formation of the known daidzein metabolites dihydrodaidzein, equol and *O*-desmethylangolensin (ODMA) and on equol- or ODMA-phenotype determination. In a study in postmenopausal women, supplemented with 160 mg isoflavones for 1 week, four of the 14 subjects (~ 29%) were classified as equol producers with average urine equol concentrations of 2915 nM [88]. All 14 subjects were considered ODMA producers, although urine concentrations varied widely. Similarly, others used a 3-day soy challenge (either providing ~ 83 mg/day daidzein or ~ 3 mg daidzein/day) to phenotype 297 obese, overweight, and normal weight individuals [86]. Around 80% of the subjects were ODMA producers and ~ 42% were equol producers. Obese individuals were 2.8-times more likely to be ODMA-non-producers. Teas et al. [91] also identified five of 17 (~ 29%) postmenopausal women as equol producers when subjects consumed a daily soy protein isolate (2 mg isoflavone/kg body weight) for 1 week in addition to placebo or seaweed (which subjects had received for the entire 7-week study period). Urinary equol excretion was enhanced when the soy protein isolate was consumed in combination with 5 g/day seaweed. In a study population of 36 young women, it was found that 26% had the equol−ODMA−phenotype, 58% equol−ODMA+, 9% equol+ODMA−, and 12% equol+ODMA+ phenotypes [84]. In phase I of this study, a soy protein formula was consumed with over 90% conjugated isoflavones (45% genistin, 40% daidzin, and 6% glycetin) equivalent to aglycone supplementation of 40 mg genistein and 20 mg daidzein daily for 4 weeks. Urinary daidzein, genistein, equol, and ODMA concentrations were measured to determine phenotype. In phase II of the same study, subjects were co-supplemented with *Lactobacillus GG* (2 × 10^10^ cfu) for 4 weeks, and this appeared to reduce total and individual isoflavone excretion by 40% either through enhancement of isoflavone deconjugation and/or blocking degradation of daidzein and genistein [84]. The stability of the equol and ODMA phenotypes over time has also been studied [85]. Phenotypes were determined by analysing equol and ODMA in urine following 3-day soya supplementation (one soy food item per day in addition to their normal diets) at two time points (T1 and T2) that were 1–3 years apart. 41% of the 92 subjects were equol producers at T1 and 45% equol producers at T2 and 90% were ODMA producers at T1 and 95% at T2. Concordance of phenotype was 82% for equol-producer phenotype and 89% for ODMA-producer phenotype. How long-term dietary habits affect soy isoflavone metabolism has also been studied [87] by comparing the plasma and prostatic fluid levels of genistein, daidzein, equol, dihydrodaidzein, and ODMA of healthy men who were considered long-term high soy consumers (30 mg or more soy isoflavones/day for at least 2 years) with those who were long-term low soy consumers (5 mg or less soy isoflavones/day for at least 2 years) at baseline and after an intervention with a soy beverage for 1 week providing between 42 and 60 mg isoflavones/serving (43% daidzein, 53% genistein, and 4% glycitein). Equol was the only metabolite affected by routine dietary habits and men who had consumed more than 30 mg isoflavones per day had 2.8 times the probability of producing equol compared to men consuming low levels of soy (28% equol producers compared to 10%). This difference was more pronounced when groups were further sub-divided into very high soy consumers (more than 50 mg/day of isoflavones). Meat consumers also appeared to be more likely to produce equol [87]. A pharmacokinetic study supplemented 11 healthy postmenopausal women with a capsule containing a isoflavone-rich soy extract (1 mg/kg bw calculated as isoflavone daidzein and genistein aglycone equivalents) measured genistein, daidzein, and various metabolites, including five microbial metabolites (dihydrodaidzein, dihydrogenistein, ODMA, 6′OH-ODMA, and equol) as well as two conjugated microbial metabolites (equol-4′sulfate and equol-7-glucuronide) [90]. Apart from equol, all of the microbial metabolites were detected in plasma and urine including the conjugates of equol. A study in Japanese postmenopausal women consuming a soy isoflavone supplement investigated the effects of short-term FOS intake on equol production [92]. Subjects were divided into equol- and non-equol producers and then assigned to groups consuming 37 mg isoflavone conjugate in capsule + 5 g sucrose as control or 37 mg isoflavone conjugate in capsule + 5 g FOS. FOS supplementation did not affect serum equol concentrations or urinary equol to daidzein concentration ratios [92]. Using a similar approach, the influence of inulin on plasma isoflavone concentrations in healthy postmenopausal women was studied using a cross-over design [89]. Twelve women consumed 2 × 40 mg per day of conjugated soy isoflavones (6 mg daidzein and 18 mg genistein as aglycone) in period one and two times 40 mg per day of conjugated soy isoflavones (6 mg daidzein and 18 mg genistein as aglycone) plus 3.66 g inulin in period two. Each period lasted for 3 weeks. Plasma 24-h areas determined at the end of each experimental period indicated that inulin increased plasma concentrations of daidzein and genistein by 38 and 91%, respectively.

Only a limited number of studies analysed IM composition or changes per se after isoflavone consumption. Supplementation with 160 mg isoflavones for 1 week in 17 postmenopausal women resulted in an increase in average relative proportion of *Bifidobacterium* and decreases in *Lactobacillus* and unclassified Clostridiaceae (analysed by 16S rRNA genes from 454 pyrosequencing). A comparison of subjects grouped into *S*-(−)equol and non-*S*-(−)equol producers showed that *Bifidobacterium, Rothia*, other Bifidobacteriaceae, and other Actinobacteria were significantly higher in *S*-(−)equol producers whereas *Roseburia* was significantly lower [88]. A total of 30 taxa significantly correlated with one to three excreted isoflavone metabolites. The effects of isoflavones on IM composition were specifically investigated in a study involving 39 postmenopausal women. All subjects received a gelified milk and cereal bar together providing 100 mg/day of isoflavones aglycone equivalents. After 30 days, the subject were split into three groups with group one receiving 100 mg isoflavones/day aglycone equivalent, group two receiving 100 mg isoflavones/day aglycone equivalent and *B. animalis* DN-173010 (10^9^ cfu), and group three receiving 100 mg isoflavones/day aglycone equivalent and FOS (7 g/day). Isoflavones alone stimulated dominant microorganisms of the *C. coccoides*–*E. rectale* cluster, *Lactobacillus*–*Enterococcus* group, *F. prausnitzii* subgroup, and *Bifidobacterium* genus. The stimulation of the *C. coccoides*-*E. rectale* cluster (which includes the isoflavone-metabolizing organisms *Peptostreptococcus productus* and *E. ramulus*) depended on the women’s equol excretion and was transient, with the exception of a prolonged bifidogenic effect. Lasting changes in the diversity of the dominant species were also observed. Urinary equol production was not different in groups receiving pro- or prebiotics in addition to the isoflavones [83].

### Data from animal studies on isoflavones

Ileal and faecal digestibility of daidzein and genistein and the extent of formation of metabolites in the intestinal tract in the ovariectomised rat, a model for postmenopausal bone loss, has been determined by measuring genistein, daidzein, and their metabolites in plasma, urine, faeces, and ileal digesta [94]. Ileal and faecal disappearance was monitored in two groups of rats that either consumed daidzein or genistein (0.026% of diet as aglycone; resulting in 10.13 µmol input per 24 h) for 4 weeks. Ileal and faecal digestibility (disappearance) was significantly lower for daidzein than for genistein. Ileal disappearance of genistein was 93% indicating that only 7% passed into the colon. The majority of the genistein remaining in the intestinal tract was unmetabolised, but small amounts of the genistein metabolite 4-ethylphenol were detected. Total digestive tract disappearance of genistein was 99.9% of intake. Ileal digestibility of daidzein was 32%, hence 68% of the total dietary intake passed into the colon. The daidzein metabolite equol was present in significant amounts in ileal digesta and was also the major metabolite excreted in faeces. Total digestive tract disappearance of daidzein was 77.5% of intake [94]. In the study by Poulsen et al. described above, equol was identified as the main daidzein metabolite in ileal digesta and faeces [94]. Both daidzein and equol were detected in plasma of the animals, but no dihydrodaidzein or ODMA. In urine, some dihydrodaidzein and 2-dehydro-ODMA were detected. On a mol/mol basis, 24-h total faecal and urinary excretion of daidzein and its known metabolites was 49.1%. 4-Ethylphenol was the main genistein metabolite identified in faeces, plasma, and urine. Small amounts of dihydrogenistein and 4-(OH)-phenyl-2-propionic acid were detected in urine. Similarly, on a mol/mol basis, 24-h total excretion of genistein and its known metabolites was 41.7%. Excretion of both daidzein and genistein was largely through urine [94]. Pharmacokinetics of dietary daidzein (20 mg) and dietary racemic equol (4 mg) were studied in ovariectomised rats [93]. Total unconjugated equol, free equol, equol monosulfate, and equol disulfate were measured in plasma and equol glucuronides were calculated. The maximum plasma concentration (*C*_max_) and time to reach it (*t*_max_) for total equol were 8815 ± 2988 nmol/l and 2.17 ± 2.91 h and 3682 ± 2675 nmol/l and 20.67 ± 4.67 h, for dietary equol and daidzein, respectively. Although the majority of equol metabolites present were glucuronide conjugates (≥ 99%), there were also low levels of equol monosulfate present [93].

None of the identified animal studies investigated changes in the IM per se. However, several studies focussed on how other substrates modulate the IM, and, in turn, affect daidzein metabolism. Three studies in mice investigated how other dietary components such as xylitol, arabinose, or rice bran oil may change the gut microbial composition which, in turn, may also affect daidzein metabolism [95–97]. In all studies, two groups of mice were fed either 0.05% daidzein in the diet alone or together with 5% xylitol, 10% rice bran oil (in this case the daidzein group also received 10% lard as control) or 2.5% arabinose in their diets. It was demonstrated that xylitol, rice bran oil, or arabinose has the potential to affect the metabolism of daidzein by altering metabolic activity of the IM and/or the gut environment. Xylitol-fed mice had more urinary equol compared to controls and the abundance of Bacteroides was significantly greater in the control than in the xylitol group [97]. Mice fed rice bran oil in combination with daidzein had significantly lower urinary amounts of daidzein and dihydrodaidzein and the ratio of equol/daidzein was significantly higher compared to the control group. The abundance of Lactobacillales was significantly higher in the rice bran group [96]. l-Arabinose-fed mice had lower urinary amounts of daidzein and the ratio of equol/daidzein was significantly compared to the control group. The abundance of *Prevotella* and Lactobacillales were significantly lower in the arabinose group [95].

In conclusion, the effects of the IM on metabolism of daidzin/daidzein are well understood. Daidzin is converted to daidzein by bacterial β-glucosidases and the predominant metabolites produced by the human IM are equol and ODMA. Among humans, 30–50% have the bacteria capable of producing equol and 80–90% harbour ODMA-producing bacteria. To date, only a few equol-forming intestinal bacteria have been identified and isolated from human faeces that harbour all the genes and express all the enzymes needed to convert daidzein to equol. However, several bacteria may work together in the metabolism of daidzein, and in vitro studies are often helpful to identify consortia of bacteria involved in the full metabolism of daidzein to equol and ODMA.

## Conclusions

The IM is comprised of at least a trillion bacterial cells per gram of faeces and most of these bacteria can be classified into 400–800 individual species. These bacteria are in an intimate balance with the host as they facilitate energy and nutrient production, by digestion of food and de novo synthesis, degradation, and bioconversion of ingested compounds and homeostasis. How the IM exerts these effects is still incompletely understood but we are starting to gain a greater understanding through the use of modern culture-independent sequencing approaches and more understanding of the broad functional activities of the IM. However, despite recent advances in methodologies, it is clear from the systematic review that there have been limited recent human studies specifically evaluating effects of IM on breakdown, absorption, and metabolism of the selected ingested dietary components with the exception of investigations on various fibres and polyphenolic compounds. Considerable data exist on the effect of ingested dietary substrates on changes in the IM composition per se and in relation with metabolism of dietary substrates using animal models (pigs, rats, mice, dogs, rabbits, kittens). However, care must be taken in extrapolating results to humans due to the inherent physiological and metabolic differences (composition of the indigenous IM, transit time, caecotrophy). Nevertheless, when taken together recent data suggest that the IM composition and related activity in response to various dietary substrates can have a significant effect on host physiology and that manipulation of the composition of the IM, particularly increasing the abundance of targeted groups, holds promise in relation with host health.

The IM has coevolved with the human host to perform functions that the host would not be able to accomplish alone. It extends the host’s metabolic capacity through, for example, substrate hydrolysis, reduction, methylation, demethylation, nitrosation, and deamination. It is evident that it can play a role in energy homeostasis. However, while differences between genera and species were noted in relation with effects on energy homeostasis, changes in phylogeny classification make it difficult to draw conclusions. Care has also to be taken regarding the usefulness or even relevance of describing the IM at the phylum level, since the “biology” and certainly most host IM interactions occur at the species/strain level. Furthermore, while faecal SCFA concentrations are widely reported, these values are the end result of a dynamic process involving production and absorption, and therefore, it is difficult to draw conclusions without more dynamic markers. In addition, interpretation of changes in SCFA production in relation with changes in “energy availability” do not take into consideration a major physiological role of SCFA produced during gut fermentation, i.e., regulation of energy intake by inducing incretins, e.g., GLP-1 which reduces food and energy intake. Therefore, the more SCFA are produced, the less energy is ingested, and less energy becomes available. Further studies are warranted that examine and differentiate energy produced by the IM via SCFA from the systemic effects of SCFA in regulating whole body or host-level energy intake and effects on energy homeostasis. Going forward, we need to apply better and more refined tools to more defined questions to make sense of diet–IM interactions in relation with energy balance. In particular, we need to ensure full characterisation of the test substrates, and methodological techniques used to characterise the IM and their metabolites to allow comparison of results across studies.

Microbial breakdown and fermentation of proteins produce ammonia, amines, phenols, and BCFA. From the limited literature retrieved in relation to high-protein intakes, it was interesting to see a greater diversity in the microbes present in the intestinal tract and higher proportion of rare genera. In general, there was an increase in Bacteroides, Fusobacterium, Proteobacteria, especially *Desulfovibrio* (*B. wadsworthia*) and *A. putredinis* and of *Bacteroides* sp. Interestingly, observed increases in *A. putredinis* and *Bacteroides* sp. correlated with higher concentrations of BCFA. Despite a global decrease in Firmicutes, *Clostridium, Eubacterium* and *Ruminococcus* were shown to increase with protein intake and *Megasphera, Selenomonas,* and *Acidaminococcus* decreased. Similarly, *Prevotella*, Archaea, and *Bifidobacterium* appear to reduce. High-protein diets tend to lead to a decrease in the total SCFA production, while valerate and BCFA concentrations are increased. Some studies have also shown increased concentrations of secondary metabolites of bile acids, particularly of DCA and higher amounts of ammonia with high-protein intakes. An inverse relationship between *Prevotella* and *Bacteroides* has previously been reported in studies examining high-protein diets and it has been suggested that a greater proportion of *Prevotella* in the IM is a marker of residence in an agrarian society, whereas predominance of Bacteroides is indicative of an inhabitant of a more industrialised society [46].

In relation to carbohydrate metabolism, certain species are known to possess genes that encode carbohydrate active enzymes and can switch readily between different energy sources in the gut depending on their availability. NSP, oligosaccharide and mucin breakdown, and fermentation by the IM lead to biomass, gas, and SCFA production which impact physiological activities, such as lipogenesis, satiety signalling, and the provision of energy to the colonocytes. Based on our review, few recent studies appear to have evaluated the effect of the IM composition and metabolism per se in relation to digestible dietary carbohydrate or fat. A diet high in carbohydrates leads to an increased production of SCFA following fermentation by the IM. Acetate, propionate, and butyrate production is generally increased, whereas valerate, BCFA, and the concentration of ammonia are generally reduced. However, no information was found during the review on the IM responsible for such changes. However, a recent study demonstrated that a high carbohydrate diet modulated faecal saccharolytic bacteria including bacteroides and bifidobacteria [44]. Studies dealing with fat intake focused on the production of methylamines. Catabolism of choline by the IM results in TMA formation which is metabolised by the liver to TMAO. Studies suggest that high-fat diets lead to increased production of both TMA and TMAO, which are possible risk factors for cardiovascular disease. Similarly, conversion of carnitine, which is high in red meat, to TMAO can occur. Concerning the composition of the IM, high-fat diets appear to lead to reduced diversity in IM. However, no information on the species impacted was found. More recent results also indicate that dietary conjugated linoleic acid metabolism in mice may be, at least, partially mediated by alterations in the IM composition and functionality [104].

In controlled human studies, variations in intake of NSP and prebiotics altered levels of specific taxa that selectively metabolise specific carbohydrate substances, such as *R. bromii and E. rectale.* In addition, levels of bifidobacteria and lactobacilli tend to increase, particularly with oligosaccharides. Few human studies were available describing the impact of RS on the IM and host physiology. *R. bromii* appears to be a key species whose growth is stimulated upon RS starch consumption both in humans and animals. Furthermore, abundance of bifidobacteria, lactobacilli, and other species belonging to the *Clostridium* cluster IV appears to be increased and leads to the production of SCFA. Although in most studies, an increase was found in the total SCFA levels, no consistency was observed in relation with the type of SCFA produced following RS consumption. Furthermore, RS intake led to an increase in the appetite promoting GLP-1 and improved colonic health.

Studies have associated increased microbial richness with diets high in fibre, vegetables, and fruits. In controlled human studies consistent data exist that show a correlation between the intake of fibre and an increase in bifidobacteria and SCFAs, in particular, butyrate. This leads to lower pH values in the intestinal tract, which is considered beneficial to the host. The type and variety of SCFA produced depended on multiple factors, such as availability of non-digestible substrate, composition of the microbial community, intestinal transit time, and colonic pH. Other indirect effects such as stimulation of GLP-1 secretion were also observed due to increases in SCFA following high-fibre diets. Regarding changes in the composition of the faecal microbiota, there are conflicting data, e.g., in one study *Bacteroides* in general were increased after fibre supplementation, whereas other studies revealed a decrease in *Bacteroides intestinalis* and *Bacteroides vulgatus.* Increased fibre intake and associated changes in metabolites and IM composition showed a positive impact on glucose and fat metabolism in humans. Of note also in relation to study results is the observation of gender differences and considerable inter-individual variation in response to dietary substrates which have implications for future study design.

There is mounting evidence that polyphenols which are abundant dietary constituents and possess antioxidant, anti-inflammatory, and anti-microbial properties may exert cardio-, chemo-, and neuro-protective effects. However, such effects are dependent on the bioavailability and bioactivity of these molecules. As most polyphenols exist in foods as esters, glycosides, or polymers, they rely on modification either by host digestive enzymes or those derived from the IM for absorption to occur. Collectively, the studies reviewed highlight the key role of the IM in the metabolism of simple and complex dietary polyphenols to produce more readily absorbable metabolites. As such, the IM are important in the potentiation of the health benefits associated with this class of compounds. Importantly, variation within the IM can have downstream consequences for the biotransformation of polyphenols and their subsequent effectiveness. Although some metabolic functions are common to a wide range of bacterial species and genera, such as deglycosylation, others are unique to a particular species or strain. For example, daidzin is converted to daidzein by bacterial β-glucosidases and the predominant metabolites produced by human intestinal bacteria are equol and ODMA [105]. Among humans, 30–50% has the bacteria capable of producing equol and 80–90% harbour ODMA-producing bacteria. However, to date, only a few equol-forming intestinal bacteria have been identified and isolated from human faeces; a consortium of four human strains (EPC4: *Enterococcus faecium* EPI1, *Lactobacillus mucosae* EPI2, *Finegoldia magna* EPI3, and a *Veillonella* sp.-related strain), *Slackia isoflavoniconvertens, Asaccharobacter celatus* and *Adlercreutzia equolifaciens*, *Eggerthella* sp. YY7918 and *L. garvieae* 20-92 [106–110].

In the polyphenol-related studies, in particular, a large amount of inter-individual variation was observed in the microbial metabolism and absorption of certain polyphenols. This variation resulted in different host exposures to bioactive compounds and could explain variation in the effectiveness of the interventions. Indeed, research by several authors [84, 89, 92, 95–97] demonstrated the potential utility of using pre- and probiotics, xylitol, arabinose or rice bran oil administration to manipulate IM interactions to alter polyphenol bioavailability/metabolism and minimize this variation. In addition, a recent study using radiolabelled (−)-epicatechin demonstrated considerable variation in the microbial processing of this flavanol between humans and rats and to a lesser extent between humans and mice. This species-dependent variation must be considered when discussing findings from rodent studies and their relevance to human health [111]. Identification of the gene families responsible for microbial conversions is increasing our understanding further. For example, two studies have looked into identification and expression of genes involved in daidzein metabolism [107, 110]. Several genes involved in the conversion of daidzein by *S. isoflavoniconvertens* have been identified and expressed demonstrating that they were encoding a daidzein reductase, a dihydrodaidzein reductase, and a tetrahydrodaidzein reductase [107]. Also, a novel NADP(H)-dependent daidzein reductase was purified from *Lactococcus* strain 20-92 [110]. Metabolic profiling is a useful tool for investigating variations in host metabolism and IM composition in response to dietary intervention [103].

In conclusion, it is evident from the systematic review that the IM plays a major and complex role in the breakdown and transformation of the dietary substrates examined. However, recent human intervention data are limited, and results observed in relation to dietary substrates are not always consistent and coherent across studies. As many of the studies reviewed were of short duration with moderate numbers of subjects, care must be taken in extrapolating results. Direct comparison of effects between studies is often hampered by lack of standardisation in methods used and differences in degree of precision in identification of the microbes involved, with some studies reporting microbial changes at phyla level while others indicating changes at species or strain level. Lack of full details and characterisation of dietary interventions and information on control of confounders applied also make interpretation difficult. Inclusion of pre-screening assessments using new tools, such as urinary markers, to differentiate responders from non-responders, may shed light on the variability of IM responses observed both in terms of composition and functionality. More long-term, well controlled, randomised, human intervention studies with adequately screened subjects and the use of culture-independent microbial technologies and novel markers are required. Nevertheless, current evidence indicates that a more thorough understanding of the role the IM plays in the metabolism of dietary substrates at precise mechanistic levels, and of the dominant members of the IM and community profiles, is essential to facilitate the development of novel dietary strategies based on the composition and physiology of the IM to improve health.
